# A Novel Near-Infrared Tricyanofuran-Based Fluorophore Probe for Polarity Detection and LD Imaging

**DOI:** 10.3390/molecules29215069

**Published:** 2024-10-26

**Authors:** Zhaojia Hang, Shengmeng Jiang, Zhitong Wu, Jin Gong, Lizhi Zhang

**Affiliations:** 1 College of Science, Gansu Agricultural University, Lanzhou 730070, China; hangzj@gsau.edu.cn; 2 School of Chemistry and Chemical Engineering, Shandong University of Technology, Zibo 255000, China; 3 School of Pharmacy, Shandong Second Medical University, Weifang 261053, China

**Keywords:** lipid droplets, polarity, near-infrared, large stokes shift, cell imaging

## Abstract

In this paper, LD-TCF, a targeting probe for lipid droplets (LDs) with a near-infrared emission wavelength and large Stokes shift, was fabricated for polarity detection by assembling a donor–π–acceptor (D–π–A) molecule with typical twisted intramolecular charge transfer (TICT) characteristics. Surprisingly, the fluorescence emission wavelength of the newly constructed probe LD-TCF was stretched to 703 nm, and the Stokes shift was amplified to 126 nm. Furthermore, LD-TCF could specifically answer the change in polarity efficiently and did not experience interference from other biologically active materials. Importantly, LD-TCF exhibited the ability to target lipid droplets, providing valuable insights for the early diagnosis and tracking of pathophysiological processes underlying LD polarity.

## 1. Introduction

It is well known that polarity is one of the most critical microenvironmental factors in biological systems, and it is the reason that the activation of multiple proteins, peptide aggregation, the maturation of nascent proteins, enzyme catalysis, protein denaturation, membrane fusion, lipid composition, apoptosis, the activation of immune responses, and signaling are all significantly relying on a balanced polarity [1,2,3,4]. Thus, monitoring changes in polarity, especially subcellular changes in polarity, is relevant for the exploration and investigation of numerous physiological and pathological processes.

Lipid droplets (LDs) are ubiquitous lipid subcellular organelles that are present in almost all eukaryotic cells, are closely related to other organelles, and have attracted much attention from researchers [5,6]. LDs are complex and dynamic multifunctional organelles that play important roles in numerous physiological and pathological processes such as energy homeostasis, innate immunity pathogenesis, autophagy regulation, membrane transport, membrane protein expression and degradation, lipid metabolism and storage, signaling, and the promotion of drug accumulation and their activation [5,6,7,8]. Increasingly, it has been suggested that the functionality of LDs is closely related to polarity, and the polarity of LDs can aptly mirror the state and function of LDs [7]. Aberrations in the polarity of LDs can perturb lipid metabolism, leading to LD dysfunction, inflammatory diseases, metabolic disorders, and even cancer [8]. Therefore, an assay of cellular polarity changes due to the accumulation of LDs could provide valuable insights for the early diagnosis and tracking of related pathophysiological processes.

Fluorescence imaging is a non-invasive, high temporal and spatial resolution, biosafe and prospective novel technology option for the in situ and real-time detection of various bioactive molecules in biological organisms, and is capable of monitoring the onset and progression of diseases at the subcellular level [9,10,11,12,13,14]. In fact, although dozens of fluorescent probes have been devised for the specific monitoring and imaging of LDs [15,16,17,18,19], polarity-sensitive near-infrared (NIR, >680 nm) fluorescent sensors with large Stokes shifts are still infrequently available, which has hampered our in-depth exploration of the intrinsic relationship between LD polarity and disease [13,14]. Nonetheless, fluorescent probes with near-infrared emissions and large Stokes shift properties have been the focus of investigation in the field of fluorescent probe generation, attributed to excellent features such as high in vivo penetration, reduced self-absorbed light interference, significant resistance to background interference, and low optical damage [20,21]. Indeed, existing LD-specific polarity probes were also potentially restricted by other drawbacks, including but not limited to difficult synthesis methods and pathways, complex molecular structures, lower sensitivity, etc. [10,11]. Therefore, the desirable fluorescent probes for imaging the polarity of lipid droplets that can simultaneously fulfill the above requirements are in imminent need of exploitation.

**Scheme 1:**
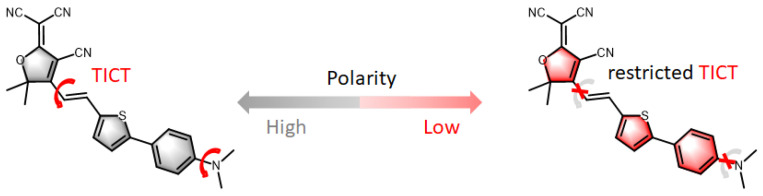
The reasonable design strategy was adopted to construct LD-TCF.

Coincidentally, it has been proposed that molecules with the donor–π–acceptor (D–π–A) structure are conducive to a polar response [22]. 2-dicyanomethylene-3-cyano-4,5,5-trimethyl-2,5-dihydrofuran (TCF), as a strong electron-withdrawing group, has been widely applied in the fabrication of D–π–A-structured probes that typically possess high-emission wavelengths [18]. In this work, on the basis of the D–π–A design strategy, TCF was selected as a fluorescent chromophore and electron acceptor. In parallel, dimethylaniline was selected as an electron donor to extend the molecular conjugation system, leading to a redshift of the emission wavelength of the entire molecule, which facilitates the imaging of biological systems. In addition, the D–π–A molecule with typical twisted intramolecular charge transfer (TICT) features was constructed by connecting the above electron donor and acceptor portions of the molecule with thiophene to form a large conjugated system, which could effectively redshift the fluorescence emission and the robustness of the thiophene, which is favorable for suppressing the loss of non-radiant energy (Scheme 1). Surprisingly, the fluorescence emission wavelength of the newly constructed probe LD-TCF was stretched to 703 nm, and the Stokes shift was amplified to 126 nm, comparing it in detail with the probes reported in Table S1 (from the Supplementary Materials) [23,24,25,26,27,28,29]. Furthermore, LD-TCF could specifically answer to the change in polarity efficiently and did not face interference by other biologically active materials. Importantly, LD-TCF exhibited the ability to target lipid droplets, providing valuable insights for the early diagnosis and tracking of pathophysiological processes underlying LD polarity.

## 2. Results and Discussion

**Scheme 2:**
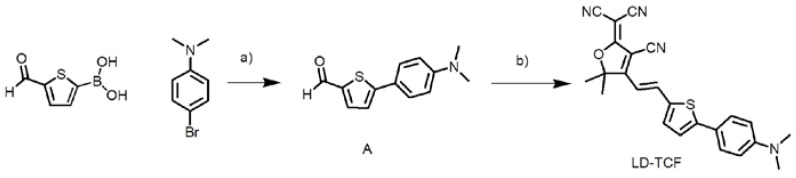
Synthesis of LD-TCF. (**a**) Pd(P(Ph_3_))_4_, K_2_CO_3_, toluene, reflux, and 24 h; (**b**) TCF, piperidine, acetonitrile, reflux, and 36 h.

The probe LD-TCF was synthesized, as depicted in Scheme 2. One of the most important intermediate compounds, TCF, was obtained in a simple one-step synthesis via malononitrile and 3-hydroxy-3-methylbutan-2-one at a high-reaction yield, according to the literature [30]. Meanwhile, another fragment constituting the probe molecule, 5-(4-(dimethylamino)phenyl)thiophene-2-carbaldehyde, was obtained by classical Suzuki-Miyaura cross-coupling with a high reaction yield [31]. However, these two molecular modules above were notably followed by the successful construction of carbon–carbon double bonds for linkage via the Kernenwenger condensation reaction in the presence of piperidine, yielding a new blue-powdered molecule of a large conjugated system at a 23% yield. Molecule LD-TCF synthesized by the above methods was subjected to structural confirmation by ^1^H NMR, ^13^C NMR, and high-resolution mass spectrometry, respectively, and these detailed data were appended to the Supplementary Materials (Figures S6–S8).

**Figure 1:**
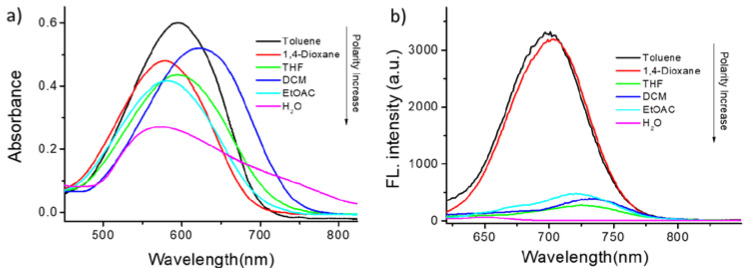
The absorption (**a**) and emission spectra (**b**) of LD-TCF (10 μM) in different solvents. λ_ex_ = 600 nm; slit = 10/10 nm.

After fabricating the probe, we first examined the absorption and emission spectra of LD-TCF in a variety of solvents representing a large range of polarity changes. As can be seen from Figure 1a, the main absorption peak of LD-TCF is focused at approximately 600 nm. For example, the maximum absorption wavelength in toluene is 596 with a molar absorptivity of 0.6 × 10^5^ L*M^−1^cm^−1^; in DCM, the maximum absorption wavelength is 620 with a molar absorptivity of 0.518 × 10^5^ L*M^−1^cm^−1^; and in ethyl acetate, the maximum absorption wavelength is 580 with a molar absorptivity of 0.417 × 10^5^ L*M^−1^cm^−1^. However, the emission spectra of LD-TCF in different polar solvents were significantly different, implying a strong dependence on solvent polarity (Figure 1b, Figure S1 from the Supplementary Materials). As the polarity of the solvent increased, the maximum emission peak from 703 nm in low-polarity 1,4-dioxane (ϕ = 0.06) to 736 nm in high-polarity dichloromethane (DCM) showed a clear positive solvatochromicity with a bathochromic change of 30 nm, and its fluorescence intensity decreased by a factor of ~8.5, which was attributed to the typical D–π–A structural features of LD-TCF that produced a clear TICT effect. Meanwhile, the Stokes shifts of LD-TCF in each of these solvents were in excess of 100 nm. By now, we had constructed a probe that simultaneously satisfied both the near-infrared emission wavelength and the large Stokes shift behavior and was able to respond dramatically to changes in polarity.

**Figure 2:**
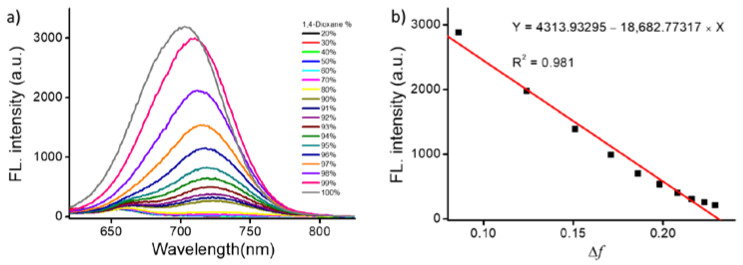
(**a**) Fluorescence emission spectra of LD-TCF (10 μM) in different ratios of 1,4-dioxane. (**b**) The linearity of emission intensity with respect to the solvent parameter Δ*f*. λ_ex_ = 600 nm; slit = 10/10 nm.

To further reveal the polarity-sensitive properties of LD-TCF and, given that the less polar 1,4-dioxane is the best mimic for biofilm structures, we assayed the fluorescence sensitivity of LD-TCF in a series of 1,4-dioxane-water systems with different ratios of 1,4-dioxane to a variety of polarities. The Lippert–Mataga polarity parameter (Δ*f*) was applied to describe the polarity of the solvent [32]. As the ratio of 1,4-dioxane increased, the Δ*f* of the mixed solution progressively decayed. As shown in Figure 2a, as the 1,4-dioxane content increased from 20% to 100%, namely, the Δ*f* decreased from 0.311 to 0.02, the fluorescence emission intensity gradually increased, and the emission wavelength was blue shifted from 726 nm to 703 nm. Notably, an excellent linear relationship (R^2^ = 0.981) was well established between the maximum fluorescence intensity of LD-TCF and Δ*f* (0.229–0.086) (Figure 2b), corroborating the high sensitivity of LD-TCF to variations in micropolarity. Furthermore, LD-TCF was tested with SDS (Sodium Dodecyl Sulfate) to verify its emission behavior in lipophilic media [15,33]. Figure S2 (from the Supplementary Materials) revealed that the emission of LD-TCF was significantly enhanced by the addition of SDS (8 mM), suggesting that it may excel when used as a probe for cellular imaging experiments.

**Scheme 3:**
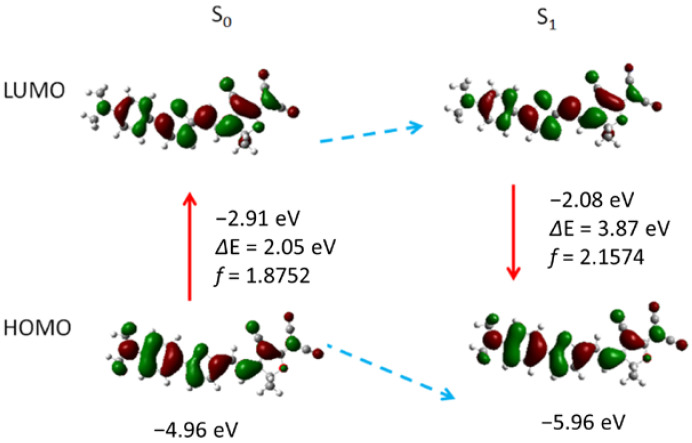
The HOMO and LUMO of LD-TCF calculated with DFT at the B3LYP/6-31G* (d, p) level using Gaussian 09.

All of the above experimental results indicate that the photophysical properties of LD-TCF are closely related to the solvent polarity, so in order to acquire a deeper understanding of the relationship between the structural characteristics and polarity-sensitive harboring activities of LD-TCF, we carried out density functional theory (DFT) calculations with the B3LYP/6-31G (d, p) basis group. As presented in Scheme 3, the highest occupied molecular orbital (HOMO) electrons were mainly localized in the TCF portion, while the lowest unoccupied molecular orbital (LUMO) electrons were mainly focalized in the dimethylaniline portion, with an energy gap of ΔE = 2.062 eV. When the molecule was excited, the intramolecular charge transfer (ICT) process occurred from the TCF unit to the dimethylaniline unit (Scheme 3). Meanwhile, the LD-TCF was conceived and engineered as a typical D–π–A system with distinct TICT characteristics. The activation of the ICT process is likely accompanied by a change in the geometry of the critical molecule, resulting in the formation of the TICT state because of the freely rotatable single bond between the thiophene and benzene ring portions of LD-TCF. As a result, in highly polar environments, the fluorescence intensity of LD-TCF was significantly reduced because the energy of the TICT state was rapidly consumed via the non-radiative relaxation pathway. In contrast, in a low-polarity solvent, LD-TCF can undergo a smaller charge separation and a weaker solvation relaxation with the surrounding solvent, emitting a stronger fluorescence.

**Figure 3:**
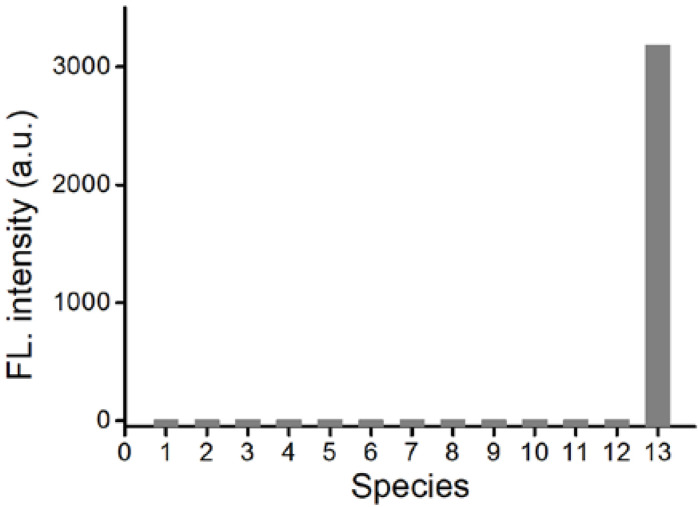
The fluorescence intensity of LD-TCF (10 μM) to various relevant analytes in the phosphate buffer. (1) Blank; (2) Cys (100 μM); (3) GSH (1 mM); (4) Hcy (100 μM); (5) Na^+^ (1 mM); (6) K^+^ (100 μM); (7) Ca^2+^ (100 μM); (8) Cu^2+^ (100 μM); (9) Fe^3+^ (100 μM); (10) HClO (100 μM); (11) H_2_O_2_ (100 μM); (12) NO (100 μM); and (13) 1,4-Dioxane. λ_ex_ = 600 nm; slit = 10/10 nm.

Subsequently, due to the fact that this D–π–A structure may also be viscosity dependent [5], the fluorescence intensity of LD-TCF was examined in solvent systems with different viscosities (glycerol/water). Although the fluorescence intensity of LD-TCF increased with viscosity, the degree of change was lower than the effect of polarity (Figure S3 from the Supplementary Materials), confirming that the sensitivity of LD-TCF to viscosity is lower than that to polarity. In addition, given the complexity of the cellular microenvironment, we validated the polar specificity of LD-TCF. As illustrated in Figure 3, the fluorescence signal feedback of the probe for 1,4-dioxane was the most remarkable compared to the relevant bioactive molecules that existed in the cell, while the signal changes induced by these other competing species were basically negligible, signifying that LD-TCF has a high specificity for polarity to keep from interfering with the results of the assay under physiological conditions. In addition, photostability is an important index to evaluate the performance of the probe, so the photostability of LD-TCF was evaluated in different polar solvents, including 1,4-dioxane, 20% 1,4-dioxane, and PBS, respectively. The experimental results reveal that the change in the fluorescence emission intensity of LD-TCF was negligible after 1200 s of continuous irradiation (Figure S4 from the Supplementary Materials), signifying that LD-TCF had favorable photostability.

**Figure 4:**
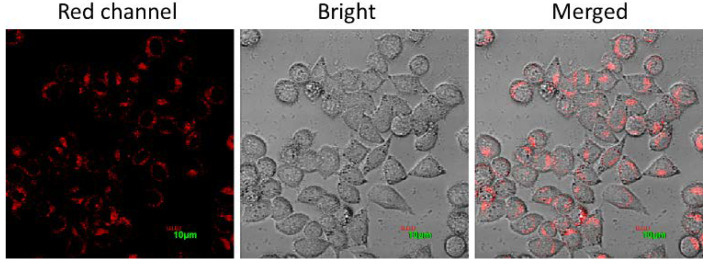
Fluorescence photo following LD-TCF (1 μM) dyeing. Cell imaging was performed by Olympus FV 1000-IX81 (Olympus, Tokyo, Japan) laser scanning confocal imaging. λ_ex_ = 635 nm, λ_em_ = 650–750 nm.

Then, we investigated the intracellular localization ability of LD-TCF by fluorescence microscope imaging. Prior to this, we first evaluated the cytotoxicity of LD-TCF using the MTT assay. Encouragingly, HeLa cells were able to sustain no less than 85% viability at a concentration of 20 µM (Figure S5 from the Supplementary Materials), indicating the relatively efficient biosafety of LD-TCF. Then, HeLa cells were incubated with LD-TCF and visualized with laser confocal microscopy. As illustrated in Figure 4, LD-TCF emitted a robust red fluorescent signature in living cells, which is indicative of the favorable permeability of LD-TCF.

**Figure 5:**
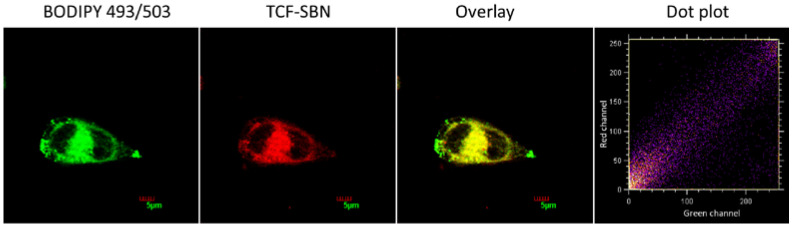
Fluorescence photo following LD-TCF (100 nM) and BODIPY 493/503 (100 nM) dyeing. Cell imaging was performed by Olympus FV 1000-IX81 laser scanning confocal imaging. Red channel: λ_ex_ = 635 nm; λ_em_ = 650–750 nm. Green channel: λ_ex_ = 488 nm; λ_em_ = 503–579 nm.

Subsequently, to further assess the probe’s sub-colocalization ability within the cell, a commercial lipid droplet targeting reagent (BODIPY 493/503 (Innochem Ltd., Atlanta, GA, USA)) and LD-TCF were employed for imaging after co-incubation. As displayed in Figure 5, the staining areas of the two kinds of stains under the red and green channels were very well matched, and the fluorescence of the two channels showed a large area of yellow color after merging. In addition, Pearson’s coefficient and overlap coefficient were as high as 0.93 and 0.92, respectively, which further indicated that LD-TCF had a satisfactory lipid droplet-targeting ability.

## 3. Materials and Methods

### 3.1. Synthesis

Compounds TCF and A were prepared according to the associated protocols [30,31].

Synthesis of LD-TCF: Under nitrogen protection, *p*-(5-formylthiophene)-*N*,*N*-dimethylaniline (43.9 mg, 0.27 mmol) and 2-dicyanomethylene-3-cyano-4,5,5-trimethyl-2,5-dihydrofuran (160 mg, 0.27 mmol) were added to a two-necked round-bottomed flask, and utilizing a syringe, an additional 1 mL of acetonitrile and 10 µL of piperidine were added to the contents. The reaction temperature was adjusted to 85 °C by reacting under vigorous stirring for 6 h. The reaction solvent was evaporated to dryness, followed by separation using column chromatography to give a blue solid (26 mg, 23%). ^1^H NMR (400 MHz, CDCl_3_, ppm): *δ* 7.69 (d, *J* = 9.0 Hz, 2H), 7.43 (d, *J* = 15.5 Hz, 1H), 7.08 (d, *J* = 3.8 Hz, 1H), 6.78–6.70 (m, 4H), 3.08 (s, 6H), 1.74 (s, 6H); ^13^C NMR (100 MHz, DMSO-*d*_6_, ppm): *δ*^13^ 177.48, 174.79, 162.39, 151.95, 150.28, 132.27, 127.40, 115.88, 113.81, 112.60, 109.48, 108.90, 98.70, 43.72, 30.73, 28.55, 26.08, 21.81; HRMS *m*/*z* = 413.1431 calcd for C_24_H_20_N_4_OS [M + H]^+^, found: 413.1427.

### 3.2. UV-Vis and Fluorescence Spectroscopy

UV-visible spectra were analyzed using a UV spectrophotometer (UV-2550, Hitachi, Tokyo, Japan). Fluorescence spectra were detected using a fluorescence spectrophotometer (F-4600, Hitachi Japan). The LD-TCF was dissolved in DMSO to prepare a stock solution, which was diluted to 10 µmol in the assays.

### 3.3. Cell Culture and Fluorescence Imaging

HeLa cells (purchased from Beijing Dingguo Changsheng Biotechnology Co., Ltd., Beijing, China) were cultured as per our prior publication [34,35]. The cells were incubated with LD-TCF (1 μM) for 30 min. For co-localization assays, 100 nM trackers and BODIPY 493/503 (100 nM) were added to the plate.

## 4. Conclusions

In summary, we have successfully formulated a novel lipid droplet-targeted and polarity-sensitive fluorescent probe, LD-TCF, using a rational D–π–A molecular design. This D–π–A type molecular structure probe presented typical TICT features, and LD-TCF exhibited strong polarity dependence, specificity, photostability, and low toxicity. Surprisingly, the fluorescence emission wavelength of the newly constructed probe LD-TCF was stretched to 703 nm, and the Stokes shift was amplified to 126 nm. Importantly, the probe had the ability to target lipid droplets. In view of its unique properties, LD-TCF will hopefully become a valuable alternative tool for cancer diagnosis and provide guidance for the further elucidation of the relationship between LDs and cancer.

## Data Availability

Data are contained within the article and Supplementary Materials.

## Supplementary Materials

The following supporting information can be downloaded at https://www.mdpi.com/article/10.3390/molecules29215069/s1. Table S1: Comparison of LD-TCF with some probes known to target lipid droplets; Figure S1: The functional relationships between fluorescence intensity and the dielectric constants of solvents; Figure S2: Relative emission spectra of LD-TCF before and after the addition of SDS; Figure S3: Emission spectra of LD-TCF with different viscosities; Figure S4: Time-dependent fluorescence changes in LD-TCF; Figure S5: MTT assay for the survival rate of HeLa cells treated with various concentrations of LD-TCF; Figure S6: ^1^H NMR spectra of compound LD-TCF in CDCl_3_; Figure S7: ^13^C NMR spectra of compound LD-TCF in DMSO-*d*_6_; Figure S8: HRMS spectrum of compound LD-TCF.
