# Supplementary material for: A Novel Near-Infrared Tricyanofuran-Based Fluorophore Probe for Polarity Detection and LD Imaging
Source: Molecules. 2024 Oct 26;29(21):5069. doi: 10.3390/molecules29215069 (PMC11547870; doi:10.3390/molecules29215069)
Supplement: Supplementary file 1 [file molecules-29-05069-s001.zip › molecules-3200837-supplementary.pdf]

Supporting Information

A novel near-infrared tricyanofuran-based fluorophore probe for polarity detection and LDs imaging

Zhaojia Hang,[a] Shengmeng Jiang,[b] Zhitong Wu,[b] Jin Gong*[, c] and Lizhi Zhang*[, b]

[a] *College of Science, Gansu Agricultural University, Lanzhou, 730070, P. R. China*

[b] *School of Chemistry and Chemical Engineering, Shandong University of Technology, Zibo, 255000 Shandong, P. R. of China*

[c] *School of Pharmacy, Shandong Second Medical University, Weifang, 261053, P. R. China*

*E-mail: zhanglizhi@sdut.edu.cn (L. Zhang) and gongjin@sdsmu.edu.cn (J. Gong)

Contents

Materials and Reagents..........S2

Table S1 Comparison of LD-TCF with some probes known to target lipid droplets..........S3

The functional relationships between fluorescence intensity and dielectric constants of solvents..........S4

Relative emission spectra of LD-TCF before and after addition of SDS..........S4

Emission spectra of LD-TCF with different viscosities..........S5

Time-dependent fluorescence changes of LD-TCF..........S5

MTT assay..........S6

^{1}H, ^{13}C NMR spectra and HRMS of LD-TCF..........S6-S7

Materials and Reagents

The various reagents and organic solvents used in the experiments were of analytical grade and were purchased from inno-chem Ltd. All reagents and organic solvents required no further purification and were used directly unless otherwise stated. The water used in the experiments was double distilled water. The progress of the reaction was monitored by observing a thin layer chromatography under an ultraviolet lamp. The thin layer chromatography used in the experiments and the silica gel (200-400 mesh) used in the column chromatography were purchased from Qingdao Ocean Chemicals. Absorption spectra were measured on UV-2550 UV/Vis spectrophotometer (Hitachi Japan), and fluorescence emission spectra were measured on F-4600 fluorescence spectrophotometer (Hitachi Japan). All fluorescence spectra data were at an excitation wavelength of 600 nm with an excitation/emission slit width of 10/10 nm. ^{1}H NMR (400 MHz) and ^{13}C NMR (100 MHz) spectra were collected by Bruker spectrometer. Chemical shift (δ) values are in ppm and tetramethylsilane is used as an internal standard. High resolution mass spectra (HRMS) were measured by Agilent 6510 Q-TOF LC/MS instrument (Agilent Technologies, Palo Alto, CA) equipped with an electrospray ionization (ESI) source. The pH was measured using a FE 20/EL 20PH meter (Mettler-Toledo Instruments (Shanghai) CO., Ltd.). Cell imaging was performed by Olympus FV 1000-IX81 (Olympus, Japan) laser scanning confocal imaging. All images were analyzed with Olympus FV1000-ASW. The cells used in the manuscript were purchased from Beijing Dingguo Changsheng Biotechnology Co., Ltd.

Table S1 Comparison of LD-TCF with some probes known to target lipid droplets.

probe	λ_{em} (nm)	Stokes shift (nm)	λ_{abs} (nm)	Ref.
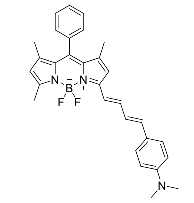	642	42	600	Anal. Chem., 2023, 95, 11785
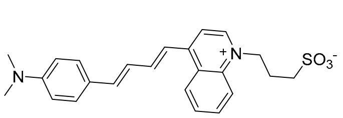	701	151	550	Analytica Chimica Acta, 2023, 1278, 341748
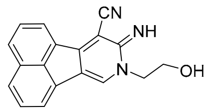	552	83	469	Biomaterials, 2018, 164, 98e105
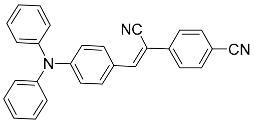	479	59	420	Dyes and Pigments, 2020, 174, 108020
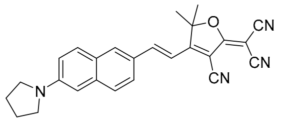	680	120	560	Dyes and Pigments, 2019, 171, 107718
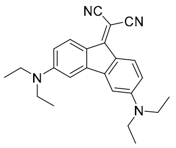	635	85	550	Sensors and Actuators: B. Chemical, 2023, 392, 134100
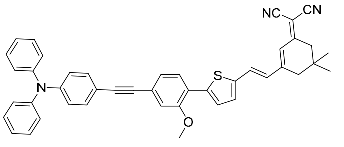	634	161	473	Biosensors and Bioelectronics, 2023, 231, 115289
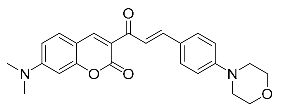	605	151	454	Chem. Commun., 2019, 55, 4703
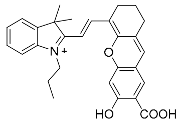	724	46	678	Chem. Sci., 2020, 11, 1617
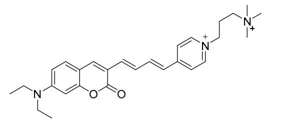	608	108	500	Anal. Chem. 2022, 94, 11089
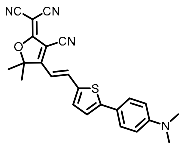	703	126	687	This work

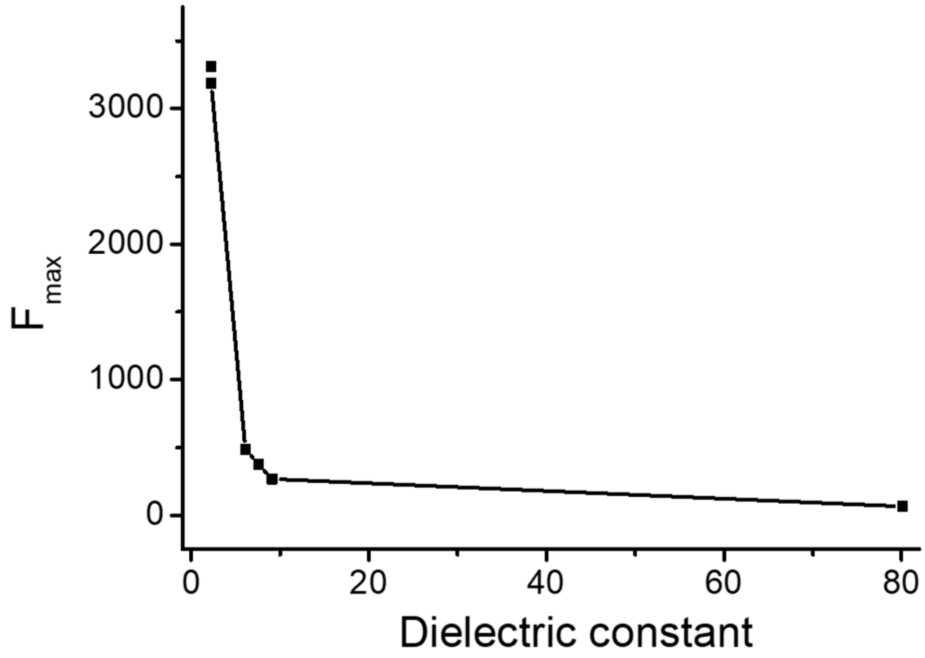

Figure S1. The functional relationships between fluorescence intensity and dielectric constants of solvents.

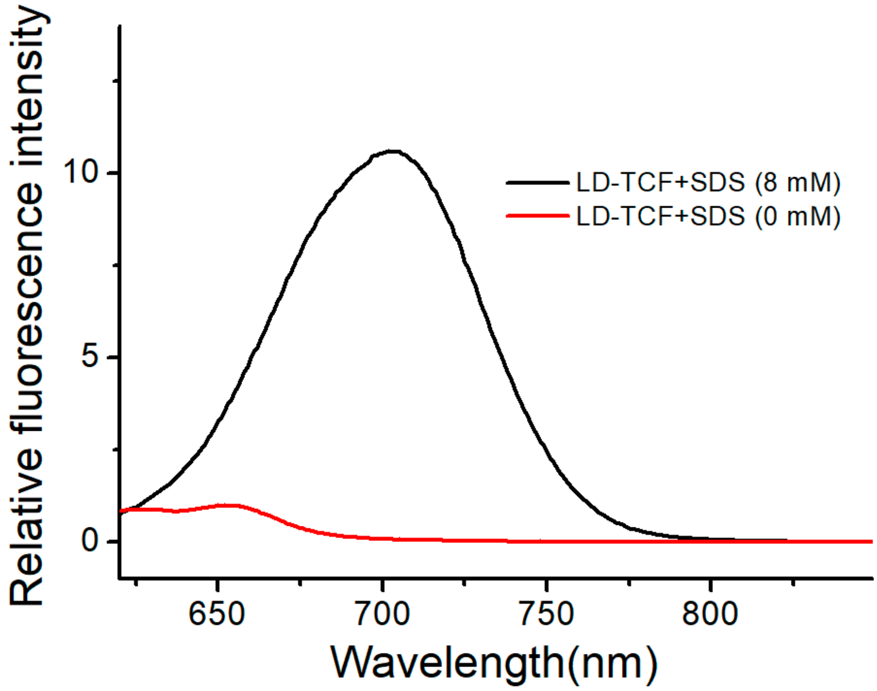

Figure S2. Relative emission spectra of LD-TCF before and after addition of SDS.

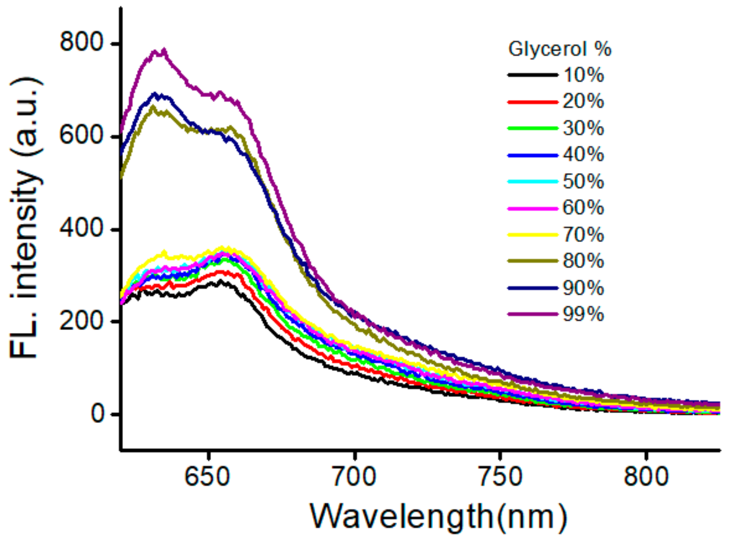

Figure S3. Emission spectra of **LD-TCF** with different viscosities (glycerol/water). λ_{ex} = 600 nm, slit = 10/10 nm.

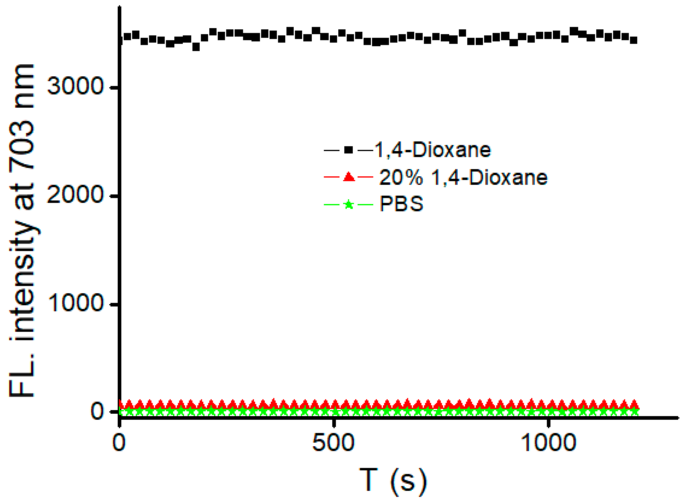

Figure S4. Time-dependent fluorescence changes of **LD-TCF** in 1,4-dioxane, 20% 1,4-dioxane and PBS with continuous illumination for 1200 s. λ_{ex} = 600 nm, slit = 10/10 nm.

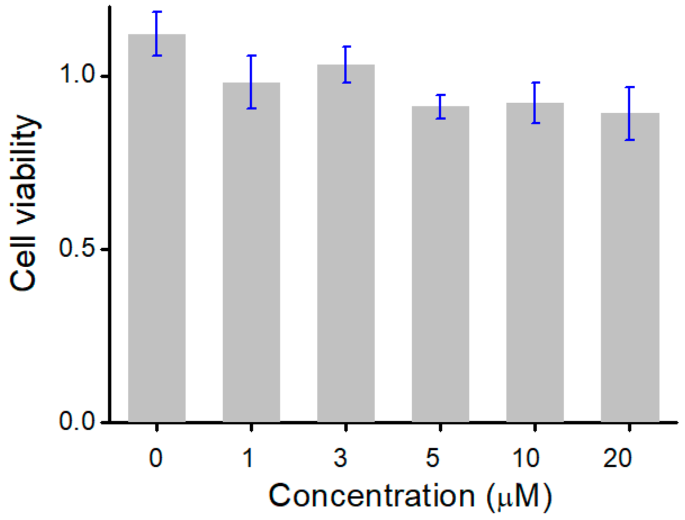

Figure S5 MTT assay for the survival rate of HeLa cells treated with various concentrations of **LD-TCF** for 24 h. Error bars represent the standard deviations of 5 trials.

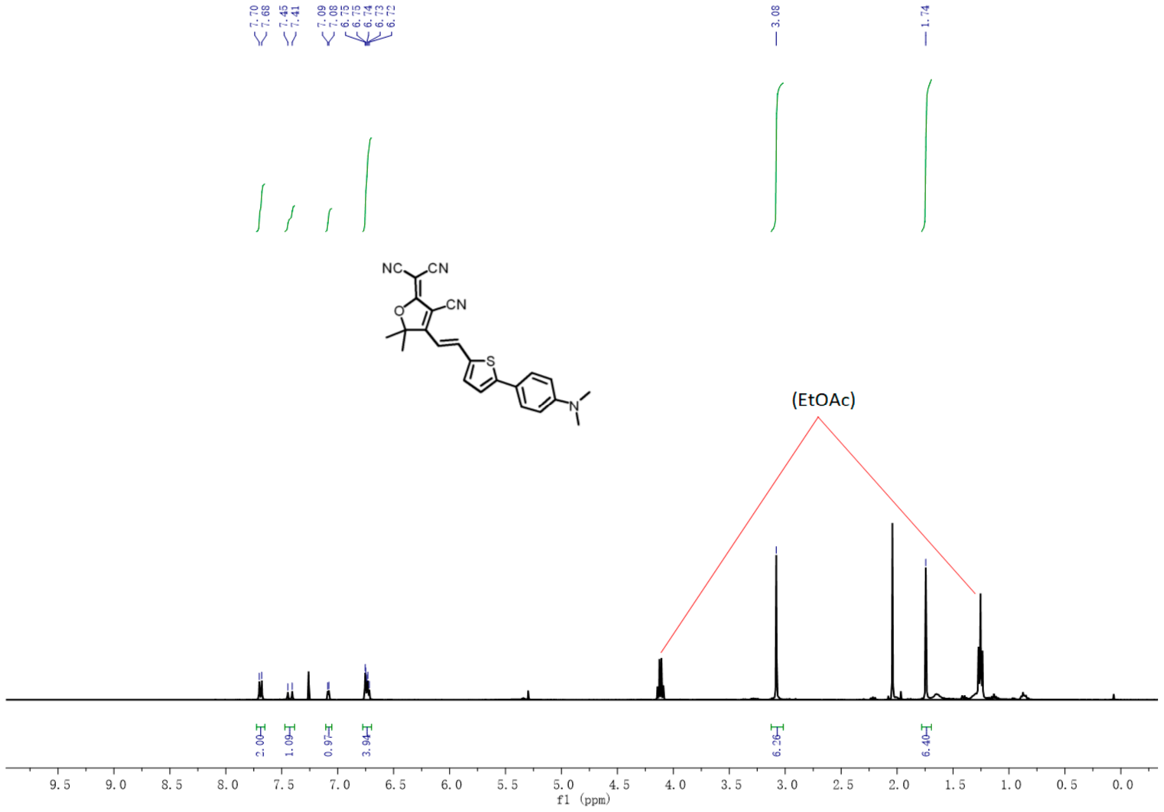

Figure S6 ^{1}H NMR spectra of compound **LD-TCF** in $CDCl_3$.

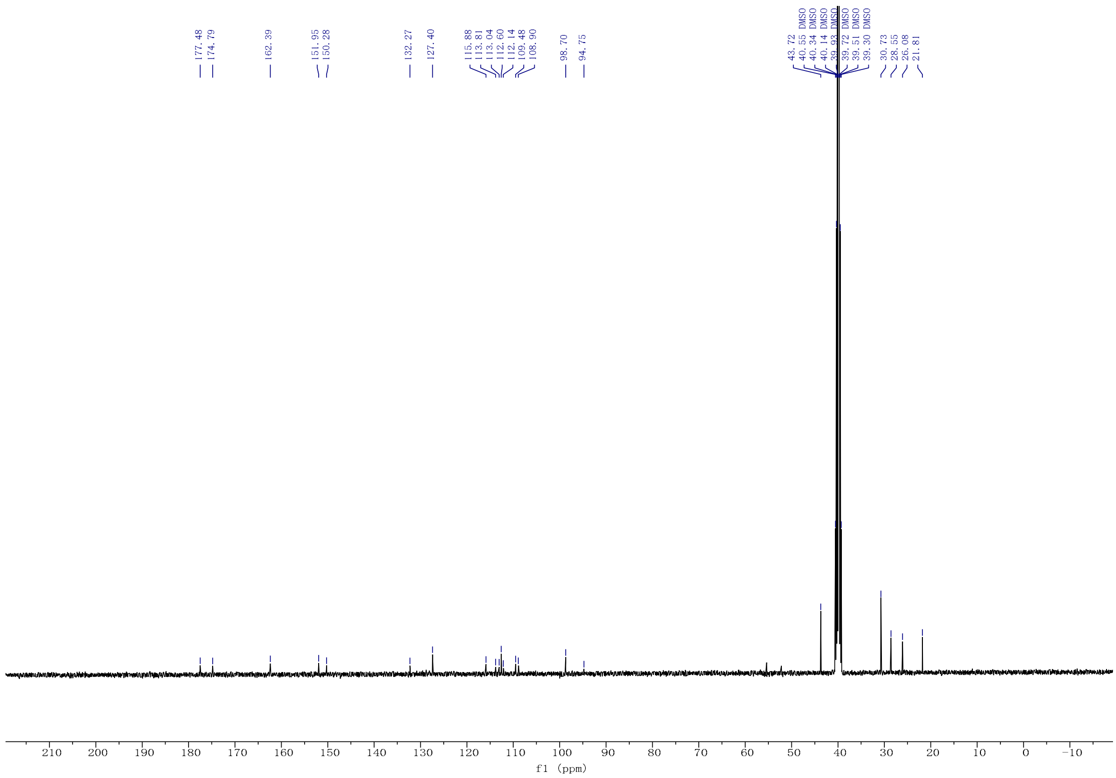

Figure S7 ^{13}C NMR spectra of compound **LD-TCF** in DMSO-d_6.

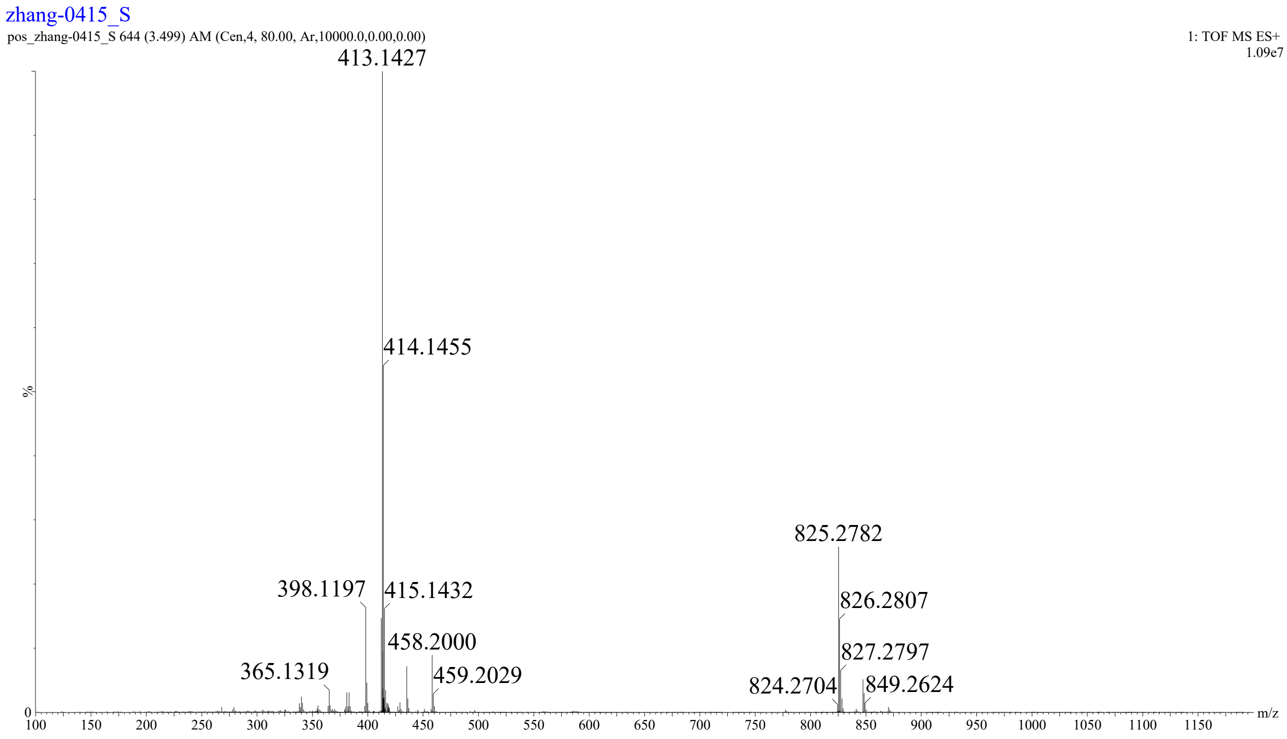

Figure S8. HRMS spectrum of compound **LD-TCF**.
